# Book Review: Ecology of the Brain: The Phenomenology and Biology of the Embodied Mind

**DOI:** 10.3389/fpsyg.2018.02174

**Published:** 2018-11-13

**Authors:** Tom Froese

**Affiliations:** Instituto de Investigaciones en Matemáticas Aplicadas y en Sistemas, Universidad Nacional Autónoma de México, Mexico City, Mexico

**Keywords:** consciousness, enactivism, 4E cognition, mind-body problem, free will

Fuchs (2018) book starts with a wake-up call. We are facing social and ecological crises that threaten the flourishing of future generations. Ideally, therefore, the sciences of the mind should help us to better understand on what basis a person can take responsible action, and thereby contribute to empowering people in their capacity to make a difference. Yet mainstream human neuroscience confronts us with the hypothesis that our self, free will, consciousness, and hence also our conscience, are nothing but internal fictions fabricated by patterns of nervous activity.

Fuchs' book is a valuable reminder of the high price of this sort of reductionism, which realizes the ideal of naturalizing the mind at the cost of leaving no theoretical room for people to genuinely make a difference for others in the world. It is a scientific worldview that implicitly legitimizes todays widespread sense of isolation and apathy. A key motivation for Fuchs is to shore up resistance against this encroachment upon our personal lifeworld, but he wisely refrains from overplaying this appeal to our conscience. The book's main contribution lies in demonstrating that doing justice to the complexities and ambiguities of human existence actually leads to a more mature cognitive science and a more coherent philosophy of mind.

Fuchs makes a sustained argument for the theory that the core purpose of mind is intelligent action in the world, which is realized by a distributed network of interactions between brain, body, and ecological environment. In this respect he is reiterating key insights from the increasingly prominent enactive approach to cognitive science, which is positioning itself as a much-needed antidote to neuro-centrism and materialistic reductionism (Thompson, 2007; Noë, 2009; Hutto and Myin, 2013; Colombetti, 2014; Gallagher, 2017; Varela et al., 2017). More importantly, the book complements recent efforts to reinterpret the role of the brain in nonrepresentational, world-involving terms (Di Paolo et al., 2017; Hutto and Myin, 2017). Actually, not too long ago critics were justified in complaining that the enactive approach has not provided an alternative framework for human neuroscience (Froese, 2015b), but Fuchs provides a detailed and compelling theory of the brain that will also appeal to less philosophically inclined neuroscientists.

His overarching thesis is that the brain is an organ of mediation and integration, rather than of mental representation and information processing. The brain has the function of helping to regulate organism-environment interactions in an appropriate manner based on acquired neuronal structures that have been shaped during the organism's history of past encounters. As Fuchs nicely demonstrates, in this way the brain also plays a central role in interactively hooking us into our social environments and thereby enabling human enculturation, and hence also the development of our higher intellectual capacities, including our capacity to make choices.

Indeed, a highlight of the book is that it does not shy away from asking the big questions of human neuroscience that have also puzzled philosophers through the ages: how is free will possible? What is the relationship between the conscious mind and unconscious matter? In addressing these questions in an interdependent manner, Fuchs develops a daring proposal that breaks with a number of ingrained beliefs about consciousness and nature. Effectively, he argues that our experience is opaquer and more constrained by its embodiment than typically assumed, and our body derives much of its spontaneous order from its animacy. Thus, by assigning priority to the person as a whole, with its dual aspects of lived and living body, the gap between subjectivity and nature becomes less prominent (Figure 1).

**Figure 1:**
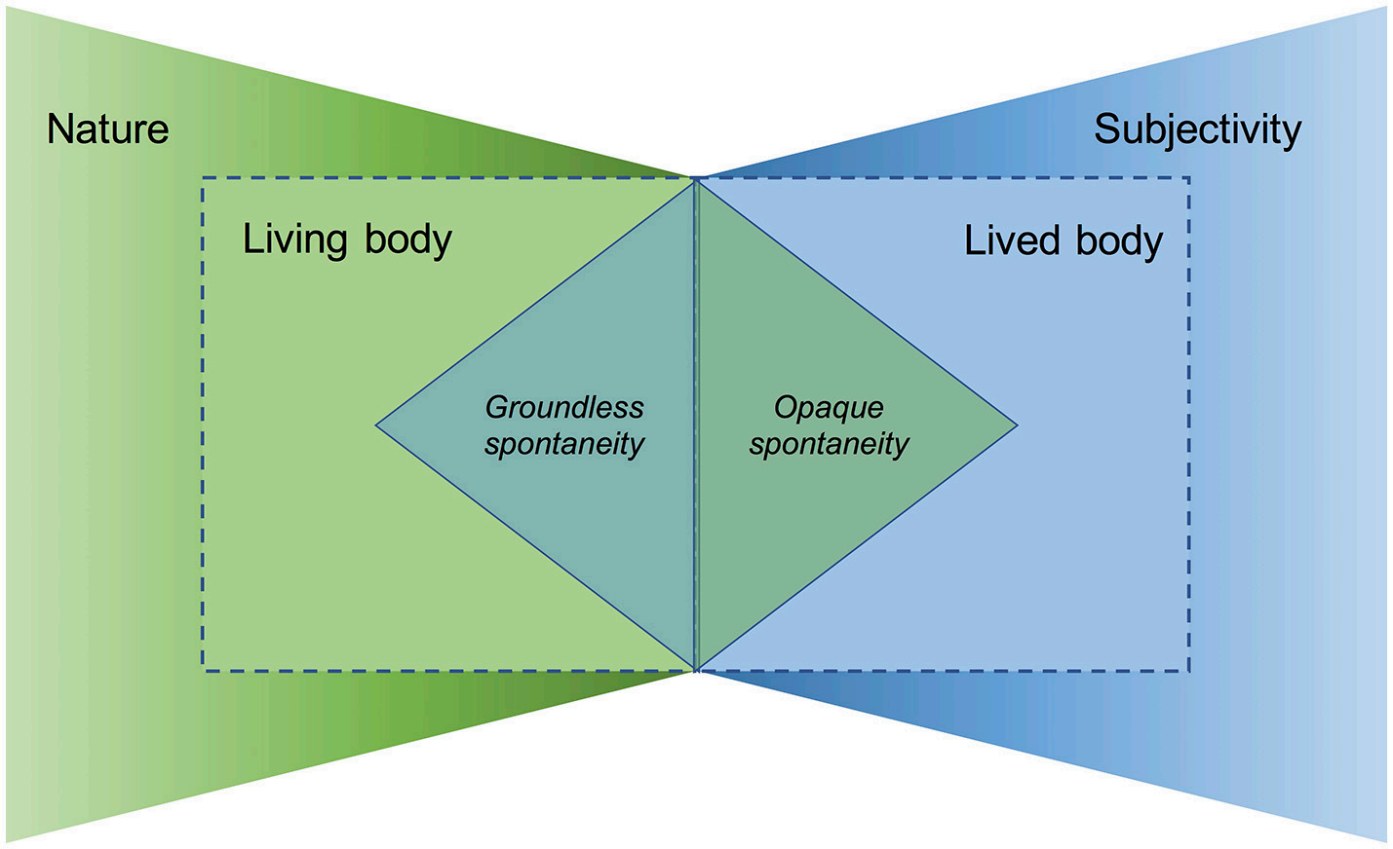
Sketch of an enactive approach to the mind-body problem. The embodiment of a person's life, in its dual aspects of lived body and living body, is the mediating interface by which the domains of subjectivity and nature come into contact with each other. Yet the two domains do not intersect directly, but rather imply each other indirectly by engendering characteristic absences. Subjectivity manifests itself in the living body in terms of groundless spontaneity, such as the emergence of order that is underdetermined by physical causes. Conversely, nature expresses itself in the lived body in terms of opaque spontaneity, such as the appearance of mental tendencies that are underdetermined by the will and whose origin and underlying basis escape our awareness.

The upshot of Fuchs' theory of dual aspectivity is that the dominant strategy of theoretically collapsing Cartesian mind-body dualism into its material aspect, and then empirically locating the mind inside the brain, is misguided and bound to fail. He proposes two complementary methodological remedies: on the side of subjectivity, we must consider not just higher-level mental processes, but the person's whole being-in-the-world, and on the side of nature, we must consider not just the brain, but the person's whole organismic embodiment in interaction with the ecological and social world. In this way Fuchs has decisively converted enactivism's original neuroscientific research program, neurophenomenology (Varela, 1996), into a long overdue neuro-physio-socio-phenomenology (Froese, 2015a).

However, Fuchs stops short of drawing the full implications of his theory. Even proponents of epiphenomenalism or identity theory could agree with these recommendations, which seem to simply broaden their quest for the neural correlates of consciousness. A much more radical theoretical and methodological shift is implied by his claim that a subject's intentional actions make a difference in the physical environment by being realized via their embodiment. Add to this that dual aspectivity entails that subjectivity and nature do not intersect directly, and Fuchs is forced to conclude that we must reject the causal closure of the physical universe. But if so, can we still scientifically investigate how subjective intentions unfold their physical effects?

I suggest, following Deacon (2012), that we should complement measuring what is physically present with measuring what is physically incomplete or even absent. Specifically, Fuchs acknowledges that if a subject's decision to act makes a difference in its own right, then the embodied realization of that action must be underdetermined by physical causes. This reveals an intriguing philosophical question that is insufficiently problematized, namely how to make room for this groundless spontaneity within nature. For instance, we need to develop stronger arguments for the claim that nature is fundamentally nondeterministic (Conway and Kochen, 2009). More practically, we need to develop ways of detecting such spontaneity, for example in terms of uncertainty, and indeed neural entropy has been found to correlate with levels of consciousness (Schartner et al., 2017).

An notable contribution of the enactive approach has been to convert the dead-end mind-body problem into a mind-body-body problem that is more tractable by phenomenological investigations (Thompson, 2007). Fuchs' book shifts the focus to the physical side of dual aspectivity, setting the stage for an expanded mind-body-body-matter problem that opens up new opportunities for human neuroscience.

## Author contributions

The author confirms being the sole contributor of this work and has approved it for publication.

### Conflict of interest statement

The author declares that the research was conducted in the absence of any commercial or financial relationships that could be construed as a potential conflict of interest.
